# Parasite density in severe malaria in Colombia

**DOI:** 10.1371/journal.pone.0235119

**Published:** 2020-06-23

**Authors:** Julio Cesar Padilla-Rodríguez, Mario J. Olivera, Bryan David Guevara-García

**Affiliations:** 1 Ministerio de Salud y Protección Social, Bogotá, D.C., Colombia; 2 Grupo de Parasitología, Instituto Nacional de Salud, Bogotá, D.C., Colombia; Instituto Rene Rachou, BRAZIL

## Abstract

**Background:**

Colombia has officially adopted the parasite density levels of severe malaria established by the WHO (>50,000 parasites/μl). These values have been inferred from areas of high transmission in Africa and are not consistent with the dynamics of low and unstable transmission in Colombia. The objective of this study was therefore to determine the parasite density values observed in patients with severe malaria and their distribution in the different ecoepidemiological regions of Colombia.

**Methods:**

A retrospective and descriptive study of confirmed cases of severe malaria was conducted in endemic areas of malaria in Colombia over the period 2014–2017. Data were collected from secondary sources of the Subnational Programs of Malaria Prevention and Control. Person, place, and time variables were selected. The official definition of severe malaria was adopted, and compliance with these criteria was determined. Univariate and bivariate analyses were conducted with absolute and relative frequency measures, and the relevant statistical tests were applied.

**Results:**

The overall parasite density values in Colombia showed a geometric mean of 5,919 parasites/μl (95% CI: 5,608–6,248). By parasite species, the values were 6,151 (95% CI: 5,631–6,718) for *Plasmodium falciparum* and 5,815 (95% CI: 5,428–6,230) for *Plasmodium vivax*. The highest parasite density values were recorded in the Amazon ecoepidemiological region (8,177; 95% CI: 6,015–11,116), and the lowest values were recorded in the Andean region (5,026; 95% CI: 2,409–10,480).

**Conclusions:**

In endemic areas of low and unstable malaria transmission in the Colombian territory, the parasite density levels observed in populations with severe malaria are lower than the officially established values. The parasite density criterion is not really a relevant criterion for the definition of severe cases in Colombia and it certainly not be used to make a clinical decision about the severity of the disease.

## Introduction

Despite marked progress in the control and prevention of malaria, the disease remains a major public health problem that contributes to morbidity and mortality, especially in children under 5 years of age [1,2]. Malaria infections can progress rapidly to severe forms of the disease involving organ dysfunction and potential death. Cases of severe malaria can be caused by *Plasmodium falciparum* or *Plasmodium vivax*, although traditionally, the progression to severe and lethal forms has been attributed mainly to infections by the former species [3]. Early access to diagnosis and antimalarial treatment can prevent complications and death from this cause [4,5]. In the 23 malaria-endemic countries in the Americas, 150,140 cases of severe forms of malaria were reported from 2000–2017, with an average of 8,341 cases per year. Severe malaria was defined as the presence of positive thick gout for *P*. *falciparum*, *P*. *vivax* or mixed infection, and with one or more complication criteria based on the malaria clinical practice guideline, included in the national surveillance system. In Colombia between 2010 and 2018, the surveillance system was notified of a total of 6,684 cases of severe malaria, most of which were caused by *P*. *vivax* and more than 95% of the patients were hospitalized and managed in critical care units. The remaining small percentage, due to geographic accessibility problems and attention opportunities, are managed at the primary level [6–8].

There is evidence of a relationship between parasitemia density and variation in the prognosis of patients with severe malaria as a function of the level of disease transmission [9]. High levels of parasitemia constitute a potential risk factor for complications and death from this cause. In endemic areas with stable and high transmission, such as Africa, complications and mortality from malaria increase with parasite densities above 10%. In contrast, in endemic areas with unstable and low transmission, such as the Americas and Asia, the risk increases with parasitemia densities much lower than 2%. However, parasitemia >20% is associated with a high risk in all epidemiological settings [3].

There is currently a gap in knowledge regarding parasite density thresholds in patients with severe malaria in endemic regions of Colombia. Although this threshold has been defined as ≥50,000 parasites/μl [5], this value is not supported by local evidence; on the contrary, it was uncritically adopted based on criteria defined by the WHO in countries with high transmission. In addition, it was not considered that the definition of hyperparasitemia may not be similar in different areas and may vary over time in the future [10].

Defining parasite density levels is important for evaluating the potential severity of the disease, the initiation of etiological treatment and the monitoring of the therapeutic response. Therefore, it is necessary to generate evidence to serve as a national reference for the diagnosis and management of severe malaria cases and to establish valid national parameters for surveillance protocols and clinical management guidelines in Colombia. All of the above efforts will contribute to and facilitate the implementation of diagnosis, treatment, investigation and response as a fundamental strategy within the elimination process that is currently being carried out in the country [11]. The objective of this study was to determine the parasite density thresholds observed in patients with severe malaria and in the different ecoepidemiological regions of Colombia.

## Methods

### Study design and data sources

A retrospective, observational and descriptive study observational was conducted based on the confirmed cases of severe malaria recorded in the endemic departments with the greatest burden of this event in the country from 2014–2017. Secondary information sources provided by the departmental and district programs for the prevention and control of malaria in Colombia were used.

### Severe malaria

The definition of a case of severe malaria established by the national malaria epidemiological surveillance protocol was adopted [12]. Cases that met the following inclusion criteria were selected: 1) history of fever, 2) positive thick drop result for malaria, 3) involvement of at least one organ and 4) available recorded data on the study variables. All cases with parasitemia <250 parasites/μl were excluded.

The criteria contained in the malaria surveillance protocol for Colombia are as follows [12]. A case of severe malaria was defined as the patient with a current or recent febrile episode diagnosed with malaria with clinical or laboratory findings that indicate serious involvement of one or more organs:

#### Cerebral malaria

Disorders of consciousness including coma, multiple seizures more than two episodes in 24 hours, prostration, or extreme weakness of the patient with difficulty or inability to sit, stand, walk without assistance, inability to feed.

#### Renal complication

Serum creatinine >1.5 mg/dL, BUN >20 mg and or urine volume <400 ml in 24 hours (adults) or urine volume <12 ml/kg of weight in 24 hours (children).

#### Hepatic complication

Presence of jaundice (total serum bilirubin >3 mg/dL) and abnormal liver function tests (Transaminases >40 IU/L).

#### Pulmonary complication or respiratory distress syndrome

Increased respiratory rate on admission, presence of pulmonary auscultation disorders such as wheezing, snoring, rales, and radiographic changes consistent with pulmonary edema.

#### Other complications should be considered

Shock, hypoglycemia (<40 mg/dL), hyperemesis, hyperpyrexia (> 39.5°C), severe anemia (hemoglobin <7 g/dL), spontaneous abnormal bleeding or disseminated intravascular coagulation, metabolic acidosis (plasma bicarbonate <15 mmol/L, base excess >-10, acidemia: arterial pH <7.35, lactate >5 mmol/L), gross hemoglobinuria or hyperparasitemia ≥50,000 asexual forms/μl of *P*. *falciparum* or in mixed malaria with *P*. *vivax* and schizontemia.

### Estimation of parasite density

The diagnosis of malaria and the level of parasite density in Colombia is made according to the guideline for laboratory surveillance of parasites of the genus *Plasmodium spp*. that was developed by the National Institute of Health. Microscopic examination of the blood smear is the gold standard in malaria diagnosis.

The method used to determine the parasite density in all cases was a quantitative method that allows estimating the level of parasitemia in the thick blood smear examination, using this simple mathematical formula: Number of parasites x 8,000 / Number of WBCs (200).

- Count parasites in relation to a predetermined number of white blood cell (WBC) count and an average of 8000/μl is taken as standard.
- 200 leucocytes are counted.
- All parasite species and forms including both sexual and asexual forms are counted together.
- If >10 parasites/100 fields are observed when counting 200 WBCs, the formula based on 200 WBCs can be applied.
- If <9 parasites/100 fields are observed when counting 200 WBCs, then the count should continue until reaching 500 WBCs and apply the formula based on 500 WBCs.
- If more than 500 parasites are observed without counting 200 WBCs, the count will be terminated when the reading of the last field is completed and the parasitemia must be calculated according to the previous formula.

### Ecoepidemiological regions

Ecoepidemiological regions were defined as receptive areas for malaria located below 1,600 meters above sea level, with geographic, ecological and entomological conditions favoring the endemic transmission of the disease [13]. Accordingly, the following ecoepidemiological regions were defined: Pacific Coast (departments of Cauca, Chocó Nariño and Valle), Amazon (departments of Amazonas, Caquetá, Putumayo and Vaupes), Orinoquía (departments of Arauca, Vichada, Meta, Casanare, Guainía and Guaviare), Sinú-San Jorge-Lower Cauca-Urabá-Middle Magdalena region (departments of Antioquia, Córdoba and Bolívar) and Andina (Huila, Risaralda and Tolima).

### Statistical analysis

A database was designed in MS Excel (Microsoft, Redmond, USA) that contained the different study variables, and quality control of the data was performed. Data analysis was conducted using Stata (release 14, Stata Corporation, College Station, TX, USA) and ArcGIS version 10.5 (ESRI, Redlands, CA) was used to produce maps. The main person, place and time variables were count and type of parasitic species; patient age and sex; country, department, district, municipality and ecoepidemiological region; and endemic and epidemic years. In the data analysis, absolute and relative frequencies were used at the national and regional levels. The p75, p50 and p25 for parasitemia were determined, and the interquartile range was calculated. Measures of central tendency were applied, including the geometric mean with 95% confidence interval. Malaria prevalence for each age group were calculated as the number of malaria case events divided by the total number of people in that age group and considering the infecting parasite species.

Graphical evaluation of the sampling distribution of the parasitemia values showed a data distribution skewed to the right. This was confirmed with the Kolmogorov-Smirnov hypothesis test (statistic: 0.1548; p<0.001). Continuous variables were analyzed using the Mann-Whitney U test. The level of significance adopted was p<0.05.

### Ethics statement

The present study met the ethical requirements established in Resolution 8430 of 1993 of the Ministry of Health of Colombia, Article 11, which establishes that studies such as the present one are risk-free and do not require approval by the Ethics Committee. Confidentiality and anonymity of the data were guaranteed.

## Results

### Population characteristics

Over the study period, 2,834 cases of severe malaria were recorded, of which 2,352 cases met the inclusion criteria. The mean age was 24 years, with a standard deviation of 18.2 (range 1–97 years). The female sex predominated with 55.7% as sex ratio of 0.79. In terms of ethnicity, 80% of the cases occurred in the Afromestizo population.

### Distribution of cases by study variables

**Fig 1:**
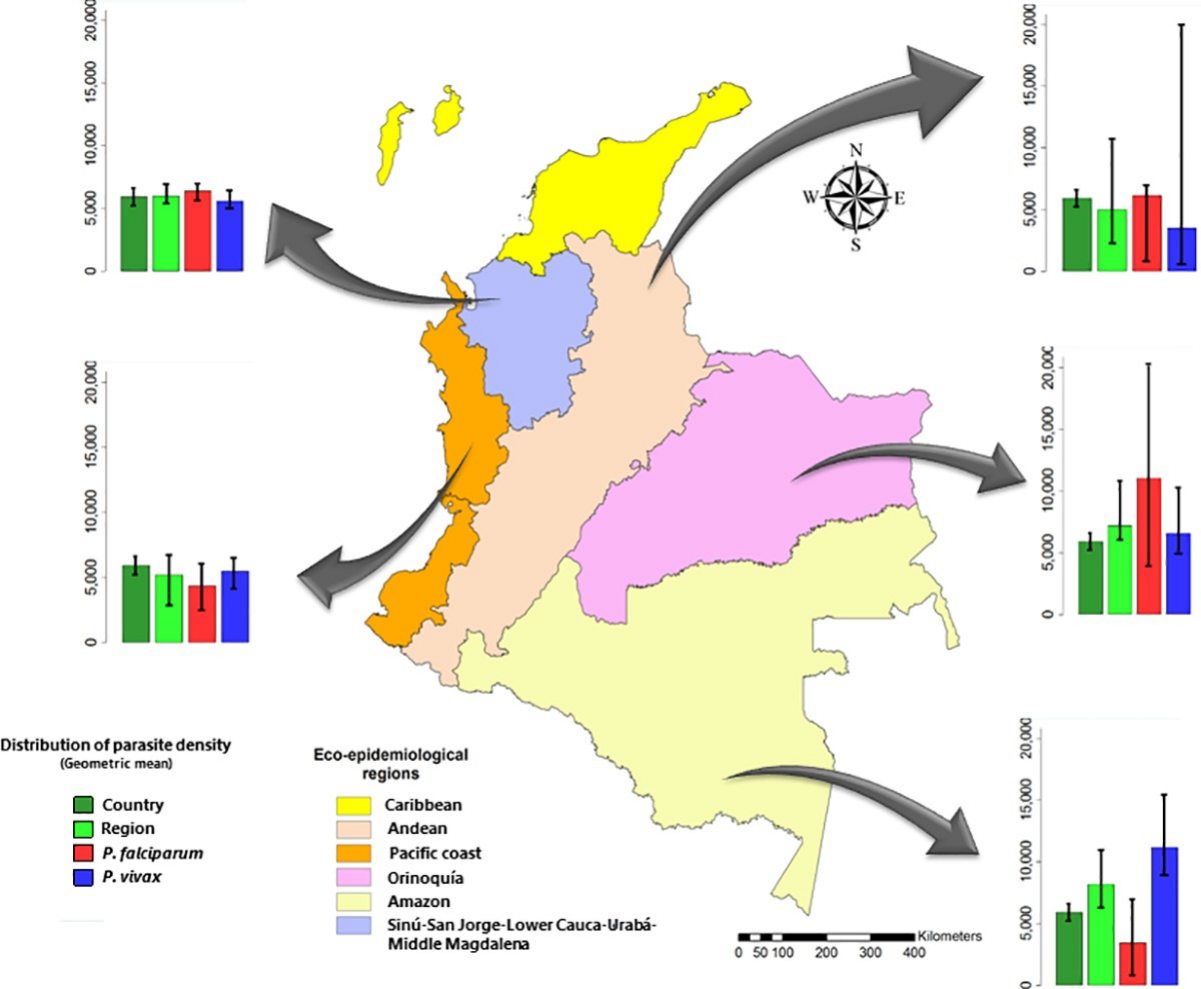
Distribution of parasite density levels in severe malaria by ecoepidemiological region and parasitic species in Colombia, 2014–2017.

The distribution of severe cases by parasite species was as follows: 1,162 (49.4%) corresponding to *P*. *vivax*; 1,090 (46.3%) to *P*. *falciparum*; and the rest to mixed forms. In terms of the distribution by ecoepidemiological region, more than 90% of the cases of severe malaria were reported in the Pacific Region with 1,728 cases (73.5%), and in the region comprised by Sinú-San Jorge-Lower Cauca-Urabá-Middle Magdalena, with 469 (19.9%) cases. The remaining 6.6% were distributed among the Amazon (88 cases, 3.7%), Orinoquía (56 cases, 2.4%) and Andean (11 cases, 0.5%) regions (Fig 1).

Regarding the distribution by year, it was observed that the largest number of cases was reported in 2016, (906, 38.5%) and the smallest number was reported in 2014 (206, 11.3%). On average, 588 cases of severe malaria were recorded per year. In terms of parasite species, *P*. *vivax* was responsible for the largest proportion of cases, with 49.4% (1162).

### Distribution of parasite density levels in severe malaria

The geometric mean for overall parasitemia in severe malaria in Colombia was 5,919 parasites/μl (95% CI: 5,608–6,248). In terms of parasite species, this value was 6,151 parasites/μl (95% CI: 5,631–6,718) for *P*. *falciparum* and 5815 parasites/μl (95% CI: 5,428–6,230) for *P*. *vivax*. The interquartile range for parasitemia was 6,294. In other words, the intermediate 50% of the data was between 2,320 (Q1) and 14,909 (Q3).

**Fig 2:**
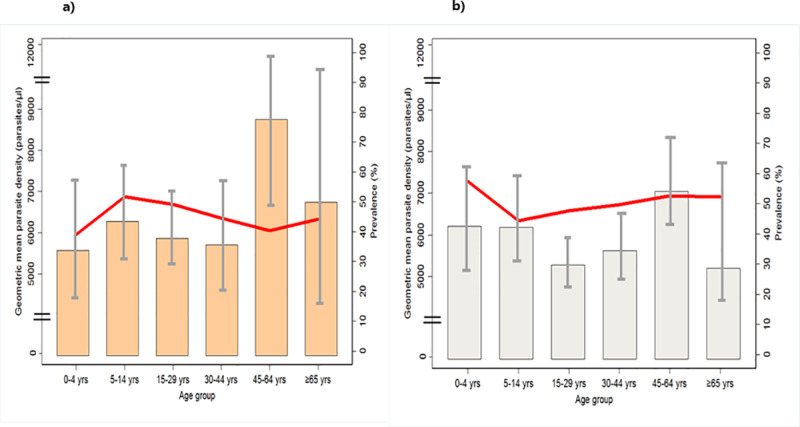
Prevalence of malaria and geometric mean parasite densities according to the infecting species. a) Age-specific prevalence of malaria and geometric mean parasite densities in *P*. *falciparum*. Line indicates prevalence of malaria. Bars indicate geometric parasite density and 95% confidence interval. b) Age-specific prevalence of malaria and geometric mean parasite densities in *P*. *vivax*. Line indicates prevalence of malaria. Bars indicate geometric parasite density and 95% confidence interval.

Regarding age, the 45- to 64 year-old group had the highest parasite density values (7,313; 95% CI: 6,158–8,684), and the 15- to 29-year-old group had the lowest (5,514; 95% CI: 5,039–6,033) (Table 1). In the age group <5 years, *P*. *vivax* infection predominated, and in contrast, *P*. *falciparum* infection was more frequent in the age group from 5 to 14 years old (Fig 2).

**Table 1:** Distribution of parasite density levels in severe malaria according to age in Colombia, 2014–2017.

Age (years)	N	Geometric mean (parasites/μl)	95% CI
<5	307	6032	5188–7014
5–14	458	6222	5507–7030
15–29	821	5514	5039–6033
30–44	420	5616	4951–6371
45–64	260	7313	6158–8684
≥65	86	5702	4188–7765

Regarding the distribution of parasitemia by ecoepidemiological region, the highest values were recorded in the Amazon region (geometric mean 8177; 95% CI: 6,015–11,116), and the lowest values were recorded in the Andean region (geometric mean 5026; 95% CI: 2,409–10,480) (Table 2).

**Table 2:** Distribution of parasite density levels in severe malaria by ecoepidemiological region and parasite species in Colombia, 2014–2017.

Ecoepidemiological region	Geometric mean (parasites/μl)					
	Overall	95% CI	*P*. *falciparum*	95% CI	*P*. *vivax*	95% CI
Amazon	8,177	(6,015–11,116)	3,463	(1,520–7,763)	11,158	(8,297–15,006)
Pacific Coast	5,206	(4,681–5,789)	4,355	(3,295–5,756)	5,495	(4,884–6,183)
Sinu-San Jorge-Lower Cauca-Uraba-Middle Magdalena	5,997	(5,622–6,398)	6,391	(5,819–7,020)	5,608	(5,125–6,137)
Orinoquía	7,207	(4,917–10,563)	11,050	(3,088–39,545)	6,568	(4,407–9,789)
Andean	5,026	(2,409–10,480)	6,152	(2,225–17,012)	3,528	(569–21,870)

Higher parasitemia was observed for *P*. *falciparum* in the Orinoquía region (geometric mean 11,050; 95% CI: 3,088–39,545) and lower parasitemia was observed in the Amazon region (geometric mean 3,463; 95% CI: 1,520–7,763). In turn, for *P*. *vivax*, higher parasitemia occurred in the Amazon region (geometric mean 11,158; 95% CI: 8,297–15,006) and lower parasitemia occurred in the Andean region (geometric mean 3,528: 95% CI: 569–21,870) (Table 2).

During the epidemic year studied (2016), the geometric mean of parasitemia recorded was 6122 (95% CI: 5,599–6,694). In contrast, densities during the nonepidemic year selected (2014) were lower at 4,101 (95% CI: 3,579–4,700). These differences in the observed values were significant between the epidemic and nonepidemic years (p<0.001) and among the ecoepidemiological regions (p = 0.045).

No differences (p = 0.299) were observed in the geometric means of parasitemia between women (geometric mean 6,214; 95% CI: 5,727–6,742) and men (geometric mean 5,696; 95% CI 5,299–6,122) or among the different age groups (p = 0.167). However, there was a significant difference between the parasite species (p = 0.039).

## Discussion

In the present study, it was possible to establish that in the active malaria-endemic areas in the national territory, which have characteristics of low and unstable transmission, the parasite density levels that were observed in the population with severe forms of malaria are lower than the thresholds established and officially recommended in Colombia. Additionally, it was possible to demonstrate the existence of variability in the distribution of the geometric means of parasitemia density in cases of severe malaria with a trend toward a predominance of low values, both overall and in the different ecoepidemiological regions of the country.

According to current guidelines and protocols applied in Colombia, one of the criteria applied to define severe malaria caused by *P*. *falciparum* is hyperparasitemia, established by a cut-off point of >50,000 parasites/μl [5,12]. These values contrast with the overall geometric mean of parasite density (corresponding to 5,919 parasites/μl [95% CI: 5,608–6,248]) and with the values reported for *P*. *falciparum* and *P*. *vivax* in the present study. The definition of hyperparasitemia is variable and depends on the level of transmission in specific ecoepidemiological scenarios. In environments with low and unstable transmission, parasitemia >2% (equivalent to >100,000 parasites/μl) has been recorded, and in areas of moderate-high transmission intensity and stability, parasitemia values >5% (>250,000 parasites/μl) have been reported [14]. Nevertheless, individuals with high parasite density may be asymptomatic, and others with low densities may present severe forms of the disease due to immune factors inherent to the host and variability in the virulence of the prevalent parasite strains, among other factors [15].

The criterion of hyperparasitemia for severe malaria caused by *P*. *falciparum* in Colombia has changed in recent decades, from 250,000 parasites/μl at the end of the last century to 100,000 parasites/μl at the beginning of the first decade and up to 50,000 parasites/μl currently, according to the threshold established by the WHO [4,16,17].

A study conducted 1988 and 1989 in the Antioquia regions of Urabá and Lower Cauca, with low and unstable transmission, in which hyperparasitemia was established as 250,000 parasites/μl, found that approximately 40% of the included cases of severe malaria caused by *P*. *falciparum* had an average parasitemia <25000 parasites/μl, which was considered low at that time [18]. Likewise, in another study conducted between 2007 and 2009 in the municipalities of Tumaco, Timbiquí, Guapi and Necoclí, where hyperparasitemia was defined as >50,000 parasites/μl, a median parasitemia of 4,880 parasites/μl was found, and 75% of the patients had less than 9,980 parasites/μl. The count was significantly higher in *P*. *vivax* infections (median = 5,280) than in infections by *P*. *falciparum* (median = 3,560) [19].

The low parasitemia thresholds recorded in the present study coincide with those reported in other endemic countries in the Americas with transmission characteristics similar to ours. In studies carried out in communities of the Peruvian Amazon, geometric mean parasitemia of 4,011 parasites/μl (95% CI: 811–9746) and 3,976 parasites/μl have been reported for *P*. *falciparum* infections, and mean parasitemia of 2,282 parasites/μl in have been reported for *P*. *vivax* infections [20,21]. Likewise, in Manaus, Brazilian Amazon, a geometric mean of 4,728 parasites/μl was reported in a series of patients with severe malaria caused by *P*. *vivax* [22].

The present study showed that parasitemia in cases of severe malaria caused by *P*. *falciparum* was slightly higher than that in cases caused by *P*. *vivax*, although no significant differences were observed between these plasmodial species. One explanation is that *P*. *falciparum* merozoites penetrate indiscriminately and rapidly replicate in young or old red blood cells of affected patients, while those of *P*. *vivax* show a preference for young cells [23]. For this reason, severe malaria caused by *P*. *vivax* is generally characterized by a lower parasitemia rate than that observed in *P*. *falciparum*, but the pathogenesis related to this clinical form is not associated with significant microvascular obstruction of vital organs. Another possible explanation is that low parasitemia may mask parasite sequestration outside the vascular system, which could explain the development of severe syndromes in patients with low parasitemia levels [24].

It is noteworthy that in this study, a defined and consistent pattern was observed in the parasitemia density of severe malaria caused by *P*. *vivax* that was very similar to the parameters observed in cases of *P*. *falciparum*. This is noteworthy because the WHO excludes this criterion from the definition of severe malaria caused by this species. In addition, the absence of this criterion reduces the specificity of the definition of severe cases caused by *P*. *vivax* [15].

In terms of demographic variables, the highest parasite densities were recorded in the 15- to 29-year age group. Such individuals are part of the economically active group of susceptible migrants from nonendemic areas moving to territories with active disease transmission. According to the 2017–2018 malaria epidemiological report, individuals in this age group were those most affected by severe forms of the disease [8,25]. This pattern has been observed in other endemic countries of the region, where a consistent trend over the years has shown that the disease predominantly affects men between 15 and 24 years of age [26]. This situation could be explained by the increased occupational exposure of this age group due to migration to endemic areas with active malaria transmission, where illegal mining, illicit crops, nonuse of individual protection measures, low immunity and varied susceptibility of this population are observed. Additionally, the majority of severe cases are associated with problems with access to timely diagnosis and treatment [27,28].

Some heterogeneity was observed in the parasite density results by endemic ecoepidemiological region; the highest densities were found in the Amazon and Orinoquía regions. Possible explanations for this finding are related to the existence of receptive areas of very low transmission with low density and high dispersion of the native population, high migration of susceptible populations and difficulties accessing health services, all of which influence the dynamics of transmission in these areas [8,29,30]. In turn, low parasite density thresholds were observed in the Pacific Coast and Upper Sinú-San Jorge-Lower Cauca-Middle Urabá-Magdalena regions. These regions are historical endemic foci whose exposed population may have some degree of immunity to the disease, which could explain the low parasitemia, in addition to better access to diagnosis and treatment [31,32].

Parasite densities during epidemic periods were higher than those during endemic periods. This finding could be explained by the dynamics inherent to the factors favoring epidemics, such as a concentration of susceptible individuals from nonendemic areas, a native population with low immunity to the infection, and a native or migrant population carrying parasites [33,34]. However, low thresholds can be found in epidemic situations, as observed in a study of an outbreak of malaria in the Emberá indigenous population, where lower geometric means of parasitemia were found for both *P*. *vivax* (geometric mean 4,858 parasites/μl; 95% CI: 4,180–5,646) and *P*. *falciparum* (geometric mean 4,777 parasites/μl; 95% CI; 2,873–7,943) [35].

The results of this study show that the parasite density criterion is not really a relevant criterion for the definition of severe cases in Colombia, since the low levels of parasitemia observed in this large number of patients with severe malaria are similar to the levels found in patients with uncomplicated malaria [36, 37]. Therefore, parasite density should certainly not be used to make a clinical decision about the severity of the disease. The findings will also allow decision-makers to make appropriate adjustments to the current definitions included in clinical practice guidelines and epidemiological surveillance protocols.

The main limitations of this study are related to the quality of the data and the secondary sources used, which may present information bias. However, the data were cleaned to ensure their reliability and validity in order to control for this bias. Another limitation was the definition of the lower limit of parasitemia of 250 parasites/μL, which was justified given that beyond this parasitemia level, cases were detected that met other criteria for severe malaria.

## Conclusion

The parasite density criterion is not really a relevant criterion for the definition of severe cases in Colombia and it certainly not be used to make a clinical decision about the severity of the disease. The parasitemia densities in severe malaria cases recorded in Colombia are low (<0.5%) and consistent with the expectation in settings with low and unstable transmission. It was found that in the different ecoepidemiological regions, the parasite densities were less than 10,000 parasites/μl, and this finding will allow the definition of specific parameters in the different endemic areas, taking into account that these parameters can vary over time and space. The parasitemia densities in severe malaria caused by *P*. *vivax* are similar to those recorded in cases caused by *P*. *falciparum* and could serve as a cut-off point for the definition of hyperparasitemia in severe malaria caused by this species. Further studies are needed to evaluate the trend of parasitemia density in severe malaria caused by *P*. *vivax* and to establish the validity of the cut-off point determined in the present study.
